# Intelligent Anti-Jamming Decision-Making Technology Based on Knowledge Graph and DQN

**DOI:** 10.3390/s25247658

**Published:** 2025-12-17

**Authors:** Dadong Ni, Xiaoqing Liu, Junyi Du, Yuansheng Wu, Chengxu Zhou, Chenxi Wang, Haitao Xiao

**Affiliations:** 1 National Key Laboratory of Complex Aviation System Simulation, Chengdu 610036, China; nidadong16@126.com (D.N.); jydu1989@163.com (J.D.); wysefan@163.com (Y.W.); 2 The Key Laboratory of Intelligent Network and Network Security, Ministry of Education, School of Information and Communication Engineering, Xi’an Jiaotong University, Xi’an 710049, China; xiaoqing@stu.xjtu.edu.cn (X.L.); zhouchengxu@stu.xjtu.edu.cn (C.Z.); wchenxi@stu.xjtu.edu.cn (C.W.)

**Keywords:** intelligent anti-jamming, knowledge graph, reinforcement learning, hierarchical reinforcement learning, two-stage decision-making

## Abstract

Recent advancements in artificial intelligence have driven significant progress in intelligent anti-jamming communications. However, existing methods still face two major limitations: reinforcement learning-based models often suffer from slow convergence, while knowledge graph-based approaches lack dynamic interaction capabilities in complex, time-varying electromagnetic environments. To address these challenges, this paper proposes a novel two-stage intelligent decision-making framework. In the first stage, an anti-jamming knowledge graph repository is constructed to enable rapid decision-making through efficient reasoning, thereby ensuring real-time responsiveness. The second stage introduces a hierarchical reinforcement learning architecture that facilitates environmental interaction for continuous model evolution and self-adaptation. By simplifying multidimensional parameter spaces into two-dimensional decision scenarios, the proposed method effectively reduces computational complexity and accelerates convergence. Experimental results demonstrate that the proposed method achieves a 4.2% increase in the anti-jamming decision success rate and a 104.8% improvement in the transmission rate compared to state-of-the-art methods. Simulation results demonstrate the superiority of the framework in both anti-jamming performance and learning efficiency, validating its practical effectiveness in dynamic electromagnetic environments.

## 1. Introduction

With the rapid advancement of wireless communication technologies, profound transformations are occurring in both lifestyle and societal operational paradigms. However, due to the inherent openness of wireless channels, communication systems are highly vulnerable to interference in complex electromagnetic environments, which severely compromises communication efficacy and reliability [1].

In practical applications, malicious jamming poses a significant threat to communication systems. By transmitting interference signals, jammers substantially degrade the signal-to-noise ratio at the receiver, thereby disrupting normal communication processes. Consequently, anti-jamming communication technologies have emerged as critical solutions for maintaining functional communication links in hostile electromagnetic environments [2]. Recent years have witnessed the intelligent evolution of jamming techniques, which now exhibit real-time sensing and adaptive adjustment capabilities. This development imposes stricter requirements on communication anti-jamming technologies and has propelled intelligent anti-jamming decision-making to the forefront of contemporary research.

Common jamming types include single-tone, wideband barrage, partial-band, multi-tone, and tracking jamming, each exhibiting distinct time-frequency characteristics that necessitate tailored countermeasures such as dynamic frequency adaptation, power control, frequency-hopping pattern optimization, and adaptive dwell-time adjustment [3]. Within this context, the rapid identification of jamming signals and real-time selection of optimal countermeasures have become pivotal for enhancing the survivability of communication systems in complex electromagnetic environments. This study explores the integration of knowledge graphs into intelligent anti-jamming decision-making to enhance commonsense reasoning capabilities and improve intelligent processing proficiency in dynamic jamming scenarios.

As jamming techniques continue evolving toward intelligence, interference signals can dynamically adapt to countermeasures implemented by communicators, rendering traditional static strategies increasingly ineffective. In this context, cognitive anti-jamming algorithms have become a research hot spot due to their ability to perceive the environment and adaptively adjust strategies. Existing research can be primarily categorized into three main types:

The first category is frequency-domain anti-jamming methods based on Reinforcement Learning (RL). These methods proactively avoid interference by selecting available channels. For instance, reference [4] employs collaborative Q-learning (QL) with wideband spectrum sensing for channel selection, but the algorithm convergence speed is slow. Reference [5] utilizes a Deep Q-Network (DQN) for online channel selection, but its strategy dimension is singular and lacks flexibility. Recently, reference [6] proposed a QL algorithm based on a Knowledge Graph (KG), which effectively improves the convergence speed by initializing the Q-table using prior knowledge. However, when facing unknown interference patterns, the decision-making capability of this method is limited by the pre-constructed KG and lacks rapid adaptability.

The second category is power-domain anti-jamming methods based on game theory. These methods establish competitive models between friendly and enemy entities and solve for game equilibrium to obtain the optimal transmission power, thereby suppressing interference in the power dimension. Related work [7,8,9] establishes effective game theory models under low interference power conditions, while other studies [10,11] further model the problem as a Stackelberg game. Although these methods achieve adaptive power adjustment, their perspective is often limited to a single power domain. When facing intelligent, rapidly changing jamming attacks, their strategic flexibility is insufficient, and the decision-making process often struggles to meet real-time requirements.

The third category is multi-domain joint anti-jamming methods, which aim to integrate multiple resource dimensions to enhance the overall effectiveness of anti-jamming. Reference [12] adopts channel selection in the frequency domain to cope with interference, modeling it as a multi-armed bandit channel selection problem, in addition to evaluating the interference levels of the channels. For channels with moderate interference, suppressive counteraction in the power domain is adopted, modeling it as a Stackelberg game model. Reference [13] proposes a multi-domain jointed cognitive anti-jamming algorithm based on the Advantage Actor–Critic algorithm that perceives Unmanned Aerial Vehicles (UAVs) as intelligent agents and decides the interference channel based on perceived environmental spectrum states. Reference [14] introduces a new communication/deception dual-mode mechanism and proposes a multi-user anti-jamming communication joint channel and power optimization scheme based on dual-mode QL. Reference [15] explores the anti-jamming problem in UAV networks under joint channel and power allocation and proposes an anti-jamming communication algorithm based on cooperative multi-agent hierarchical QL. However, the above-mentioned algorithms all utilize RL. If the action space is too large, the convergence speed is slow.

In summary, although existing research has made significant progress in anti-jamming decision-making, there are still notable shortcomings when dealing with complex electromagnetic environments, especially in terms of rapid decision-making capability. Specifically, the limitations of existing methods can be attributed to the following three points: (1) Insufficient flexibility of single-domain anti-jamming strategies and limited decision-making speed, (2) the contradiction between the decision-making dimension and algorithm convergence speed faced by multi-domain joint schemes, and (3) the lack of rapid cognition and adaptive decision-making capability for unknown or complex interference patterns.

To address the aforementioned challenges, this paper proposes a two-stage intelligent anti-jamming decision-making framework integrating knowledge graph (KG) and hierarchical reinforcement learning (HRL). This framework aims to combine knowledge-based fast reasoning with learning-based deep optimization, expecting to significantly enhance the speed and effectiveness of UAV anti-jamming decision-making in complex electromagnetic environments. The main contributions of this paper include the following:

1. A two-stage intelligent anti-jamming decision-making framework is proposed. This framework not only ensures the rapidity and timeliness of decisions through the knowledge graph but also achieves efficient interaction with the environment through hierarchical reinforcement learning, promoting the model’s self-evolution and the dynamic evolution and expansion of the knowledge graph.
2. In response to these challenges, we propose a two-stage intelligent anti-jamming decision-making framework that integrates a knowledge graph with hierarchical reinforcement learning. This design decouples the complex multi-dimensional parameter joint optimization problem into upper- and lower-layer two-dimensional parameter optimization sub-problems, thereby significantly improving the convergence speed and stability of the DQN algorithm.
3. Through extensive simulation experiments, the performance of the proposed architecture is systematically verified and compared. The experimental results demonstrate the superiority of the proposed method in terms of anti-jamming effectiveness, decision-making speed, and algorithm convergence.

The rest of this paper is organized as follows. Section 2 provides a brief description of the methodology and the system model under consideration. Section 3 elaborates on the proposed two-stage intelligent anti-jamming decision-making framework, detailing KG-based rapid decision-making and DQN-based dynamic decision-making with a hierarchical architecture. Section 4 presents the experimental settings and a comprehensive analysis of the results, comparing the performance of the proposed method against that of several baseline algorithms. Finally, Section 5 concludes the paper by summarizing the key findings and suggesting directions for future research.

## 2. Methods

**Figure 1:**
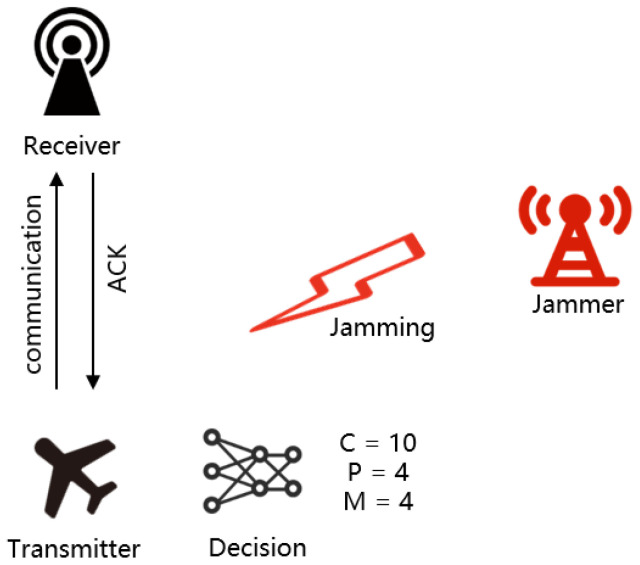
System model.

In UAVs, decision-making is categorized into transmitter-side decisions and receiver-side decisions. Transmitter-side decisions refer to a set of strategic selections made by the transmitter prior to data transmission based on factors such as current network conditions, channel quality, system resources, and communication policies. Receiver-side decisions, on the other hand, are made by the receiver regarding aspects such as channel selection and transmission power. The objective of these decisions is to optimize the efficiency, reliability, and power consumption of data transmission. In the system model proposed in this paper, transmitter-side decisions are employed. A schematic diagram of the single-UAV communication system is illustrated in Figure 1. The main components of the communication network include one UAV, a base station, and one jammer. The system model comprises C channels, P power levels, and M modulation types.

The UAV, acting as the starting point of the communication link, is responsible for transmitting data and sending information to the receiver. The UAV first performs spectrum sensing on the current communication environment to assess the state of available channels. This step is crucial, as it enables the UAV to detect which channels experience the least interference, thereby allowing it to select the optimal channel for data transmission. This paper employs the energy detection method for spectrum sensing. Energy detection is a commonly used technique in spectrum sensing to determine whether a signal is present in a specific frequency band. It does not rely on any prior knowledge of the signal, making it widely applicable in cognitive radio spectrum sensing.

After receiving the signal from the UAV, the base station not only decodes and verifies the data to confirm the integrity and accuracy of the information but also sends an acknowledgment signal back to the UAV upon successful reception. This acknowledgment signal contains feedback on the quality of the received signal, such as the signal-to-interference-plus-noise ratio or Bit Error Rate (BER). Based on this feedback, the UAV further adjusts its transmitter-side decisions to adapt to the current network environment and maximize the performance of the communication link.

## 3. System Model

**Figure 2:**
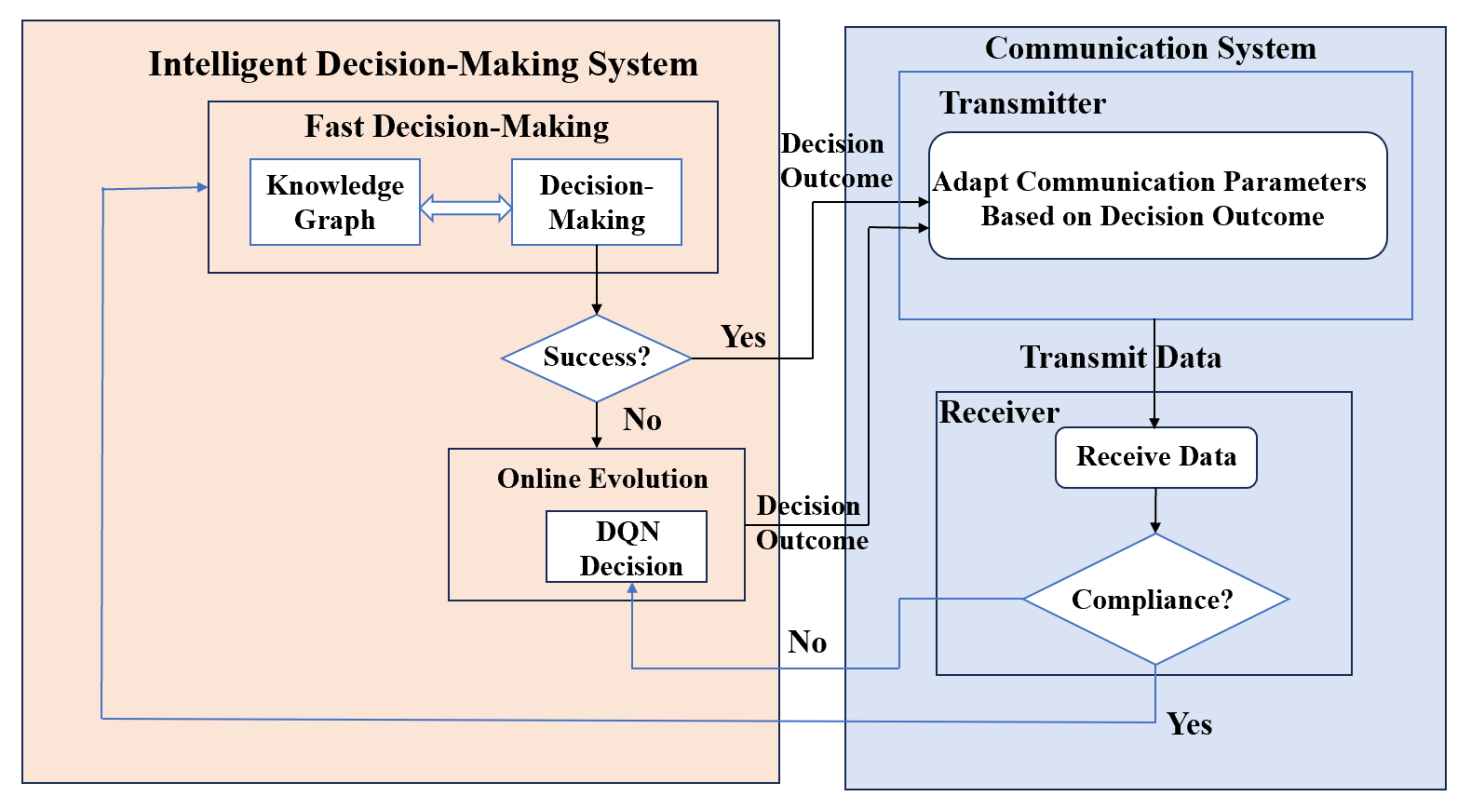
Schematic of the anti-jamming decision model.

The UAV adopts a transmitter-side decision-making strategy to select appropriate channels, modulation schemes, and transmission power to ensure efficient data transmission. As shown in Figure 2, this paper employs a two-stage anti-jamming intelligent decision-making method based on Knowledge Graph-Deep Q-Network (KG-DQN). First, a KG-based fast decision-making algorithm is utilized for initial decision generation. If a decision is successfully made, the transmitter adjusts the communication parameters and transmits the data to the receiver. If unsuccessful, a DQN-based two-layer anti-jamming decision-making method is adopted, and the transmitter adjusts the communication parameters according to the resulting decision. The receiver acquires the data transmitted by the transmitter and evaluates the BER of the received signal. This result is then fed back to the transmitter. If the outcome meets predefined criteria, it is stored in the KG repository; otherwise, the DQN-based decision-making algorithm continues to iteratively generate new decisions.

The DQN-based decision-making algorithm leverages a DQN to guide the UAV’s decision-making process, enabling it to autonomously learn how to select optimal actions in complex electromagnetic environments. This allows the UAV to maintain high-quality communication under various jamming conditions. Through this approach, the UAV can not only adapt to and identify multiple jamming patterns but also dynamically adjust its communication strategies to sustain stable and clear signal transmission, thereby enhancing the reliability and efficiency of its mission execution.

To enable rapid and informed decision-making, we first construct an anti-jamming knowledge graph repository, as detailed below.

### 3.1. Rapid Anti-Jamming Decision-Making Using KG

#### 3.1.1. Knowledge Graph Database Construction

The effectiveness of the KG construction determines its applicability, making correct construction particularly critical. The process of building a KG primarily involves the following aspects.

Data Sources: The KG is composed of a schema layer and a data layer. The schema layer corresponds to ontologies, while the data layer corresponds to instances. As abstract concepts, ontologies are used to describe relationships, rules, and classifications among various entities. These ontologies are stored in ontology models, which include elements such as jamming pattern categories. The data layer is formed through knowledge extraction and is mapped to the ontology model, representing concrete instances of the ontologies [6]. In the data layer, knowledge is represented in the form of triples: “entity (attribute)–relation (attribute)–entity (attribute)”.

The primary data of the KG comprise common jamming signals, common jamming strategies, the characteristics and attributes of jamming patterns, and the decision-making parameters of jamming strategies.

Knowledge Extraction: Given the relatively small scale of data in the field of communication anti-jamming, anti-jamming knowledge—including jamming patterns, jamming characteristics, jamming pattern attributes, anti-jamming strategies, and decision parameters—is manually extracted from unstructured data such as literature and research reports. The following entities can be identified:

Regarding jamming patterns, the entities include a single-tone jamming signal, multi-tone jamming signal, partial-band jamming signal, wideband barrage jamming signal, tracking jamming signal, sweep jamming signal, and high-speed collision jamming signal, among others. Regarding anti-jamming strategies, the following can be obtained: dynamic power adaptation, dynamic frequency-hopping set adaptation, dynamic dwell-time adaptation, dynamic modulation and coding parameter adaptation, and an increased hopping rate.

Relationship extraction: Relationship extraction involves extracting semantic relationships between entities from textual data and representing them in a structured form. Since our goal is to improve the accuracy and speed of anti-jamming decision-making by constructing a KG related to communication anti-jamming, the correspondence between jamming patterns, jamming characteristics, and anti-jamming strategies is crucial. Establishing correct correspondences enables the selection of optimal anti-jamming strategies during decision-making. The relationship template between jamming patterns and anti-jamming strategies is “Jamming Pattern—Anti-Jamming Strategy is— Anti-Jamming Strategy”.

Attribute extraction: Attribute extraction involves retrieving attribute information of entities from data such as text. Attribute extraction, together with entity extraction and relationship extraction, constitutes the core of knowledge extraction. In this study, manual extraction methods are primarily used.

Using the template of “Jamming Pattern— Has Attribute—Jamming Pattern Attribute”, results such as “Wideband Barrage Jamming—Has Attribute—Jamming Power” can be obtained.

Knowledge Representation: Since this study employs a manual approach for knowledge extraction, it avoids the ambiguity and redundancy issues that may arise from using machine learning-based extraction methods. Therefore, the knowledge fusion step can be omitted.

This study primarily uses the Resource Description Framework (RDF) to represent the KG. The RDF proposes a simple binary relational model to represent semantic relationships between entities, using a collection of triples to describe entities and their relations. Triples are used to express relationships between entities or to specify the attribute values of a particular entity.

Knowledge Storage: Neo4j uses the Cypher query language, which features a concise and intuitive syntax. Cypher not only allows for expressive representation of complex graphs but also enables efficient querying. By searching for paths between nodes, it is possible to retrieve information about all nodes related to a specific node. Even with large volumes of data, Neo4j can perform queries rapidly, which aligns with our goal of making quick decisions in response to jamming signals.

Given the substantial amount of data involved in constructing the KG, Python 3.8.18 is used to input pre-written statements into the Neo4j 5.26.0 software, facilitating easier maintenance in later stages.

For instance, to establish a relationship between nodes—for example, indicating that “Dynamic power adaptation” is a strategy applicable to “Single-tone jamming”—the statement CREATE (jam1)-[:hasAntiJammingStrategy]->(strategy2) can be used to create this connection.

**Figure 3:**
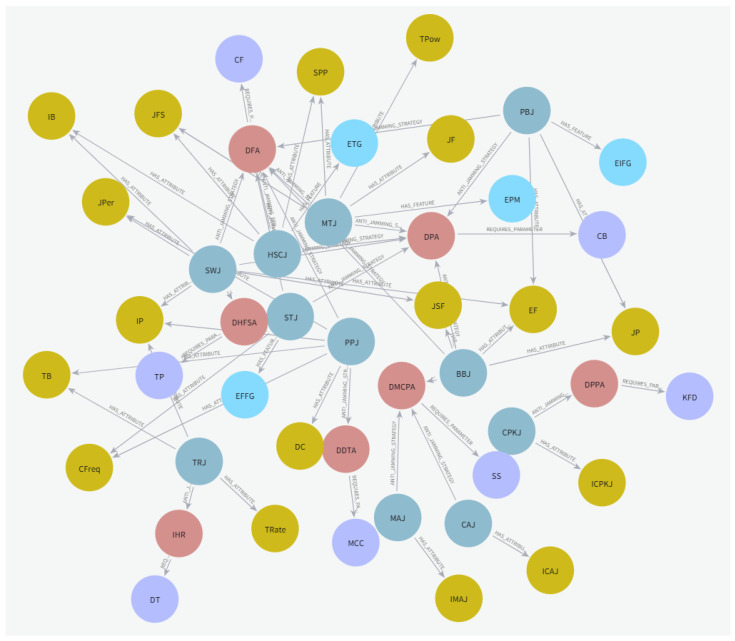
Knowledge graph.

Finally, we constructed an anti-jamming KG as depicted in Figure 3. It encompasses five node types: jamming patterns, jamming characteristics, jamming pattern attributes, anti-jamming strategies, and decision parameters. The graph includes four corresponding relationships: between jamming patterns and jamming characteristics, between jamming patterns and jamming pattern attributes, between jamming patterns and anti-jamming strategies, and between anti-jamming strategies and decision parameters. To enhance the visualization clarity of the knowledge graph, abbreviated labels were employed for all nodes and relationships. All abbreviations were designed to be unique and semantically meaningful. Table 1 provides the complete abbreviation mapping used throughout this study.

With the KG constructed, we now describe how it supports rapid anti-jamming decision-making in both known and unknown jamming scenarios.

#### 3.1.2. Decision-Making

When the jamming pattern is known, the corresponding anti-jamming strategy can be directly retrieved, since information such as jamming patterns and their corresponding countermeasures is already stored in the KG. By using the jamming pattern as the head entity and the anti-jamming strategy as the relation, the appropriate strategy can be efficiently identified, enabling rapid decision-making and ensuring real-time responsiveness.

In cases where the jamming pattern is unknown and, thus, no corresponding head entity exists for direct retrieval, the time-frequency characteristics of the jamming signal are first extracted to determine its category. The corresponding anti-jamming strategy is then derived based on this classification, completing the decision-making process.

We primarily identify jamming signals by assessing the existence of characteristics such as power margin, frequency gap, and time gap.

**Power Margin:** To determine whether a power margin exists, it is necessary to calculate the jamming-to-signal ratio (JSR), as shown in Equation (1):

(1) $$JSR=\frac{P_{j}}{P_{s}}\;,$$

where $P_{j}$ denotes the power of the jamming signal and $P_{s}$ represents the received power of the target communication signal at the receiver. When the JSR is greater than 0 dB, the jamming signal can effectively interfere with the communication signal; otherwise, effective jamming cannot be achieved. This condition is referred to as the effective interference-to-signal ratio threshold [16]. If the jamming power meets or exceeds this threshold, no power margin exists; otherwise, a power margin is present.

**Frequency Gaps:** To determine the presence of an instantaneous frequency gap, it is necessary to calculate both the frequency gap between the interfering signals and the frequency gap between the starting frequency of the communication band and the first interfering signal. The largest frequency gap among these is then identified. If the largest frequency gap is not smaller than the instantaneous bandwidth of the communication signal, an instantaneous frequency gap is considered to exist. Conversely, if the largest frequency gap is smaller than the instantaneous bandwidth of the communication signal, no instantaneous frequency gap exists.

If all frequency gaps are not smaller than the instantaneous bandwidth of the communication signal—that is, even the smallest frequency gap is no less than the instantaneous bandwidth—then a full-time instantaneous frequency gap is considered to exist. Otherwise, no full-time frequency gap exists. The formula for calculating the frequency gaps between interfering signals is shown in Equation (2) below.

(2) $$W_{i}=\left\{\begin{array}{cc} f_{i+1}-f_{i}-\frac{B_{i+1}+B_{i}}{2}\;, & f_{i+1}>f_{i}+\frac{B_{i+1}+B_{i}}{2}\;;\\ 0\;, & f_{i+1}<f_{i}+\frac{B_{i+1}+B_{i}}{2}\;. \end{array}\right.$$

$W_{i}$ denotes the frequency gap, $f_{i}$ indicates the center frequency of the interfering signal, and $B_{i}$ denotes the bandwidth of the jamming signal.

**Time Gaps:** To determine the presence of a time gap, the following condition must be satisfied: if, during a specific time period, no interfering signal is present or the JSR is sufficiently low—indicating the existence of a power margin—then the communication signal remains unaffected by interference, confirming the presence of a time gap.

Upon determination of the anti-jamming strategy, it is necessary to define the corresponding decision parameters. The parameters for anti-jamming decisions are essentially the time-frequency parameters that ensure normal communication.

The anti-jamming strategies incorporated in the KG constructed in this study include dynamic frequency adaptation, dynamic power adaptation, dynamic modulation and coding parameter adaptation, increased hopping rate, dynamic frequency-hopping set adaptation, dynamic dwell-time adaptation, and dynamic protocol parameter adaptation. Therefore, this paper focuses only on the decision parameters corresponding to these strategies.

Time-domain decision parameters: For dynamic dwell time adaptation, the decision parameter is the dwell time. The parameter selection method involves maintaining radio silence during time slots affected by interference and resuming communication during interference-free slots.

Frequency-domain decision parameters: Frequency-domain anti-jamming strategies include dynamic frequency-hopping set adaptation and dynamic frequency adaptation.

For dynamic frequency adaptation, the decision parameter is the available communication frequency band. If the gap meets the bandwidth requirements of the communication system, it can be selected as the operational frequency band for dynamic frequency adaptation.

For dynamic frequency-hopping set adaptation, the decision parameters are the frequency-hopping set and the dwell time. After calculating instantaneous frequency gaps, for each dwell time, the instantaneous frequency gaps that satisfy the communication bandwidth requirements are identified. These are then organized into a frequency-hopping pattern, which serves as the frequency-hopping set for this strategy. For example, in the case of linear sweep jamming, unaffected frequency gaps may be selected as available bands, which are then divided into a frequency-hopping set according to the communication bandwidth.

Power-domain and modulation/coding decision parameters: In practical applications, it is necessary to first select the modulation order and channel coding rate based on requirements, then determine the reliable transmission power threshold. Therefore, these two parameters are studied together.

The modulation order and channel coding rate are determined based on communication requirements: The lowest-order modulation and minimum channel coding rate are used for minimal power consumption, while the highest-order modulation and maximum channel coding rate are adopted for the maximum transmission rate.

The transmission power must be greater than the sum of the jamming signal power and the signal-to-interference ratio threshold required by the receiver under the chosen modulation and coding scheme.

Decision parameter for increased hopping-rate strategy: The decision parameter is the hopping rate. In practice, this often involves selecting from predefined fixed levels. The decision method involves increasing the hopping rate when tracking jamming is detected.

Decision parameter for dynamic protocol parameter adaptation: The decision parameter is the keyframe transmission delay. Similarly, this parameter typically has predefined fixed levels in actual applications. The decision method involves adjusting the transmission delay of key frames—either increasing or decreasing it by one level—when key frames of the carrier sense a multiple-access protocol.

### 3.2. DQN-Based Anti-Jamming Dynamic Decision-Making

Addressing the vulnerability of individual UAVs to various types of jamming and considering constraints such as the transmission rate and energy consumption, this paper investigates anti-jamming techniques for UAVs in complex electromagnetic environments. By integrating jamming countermeasures across the frequency, time, power, and code domains, a multi-domain collaborative anti-jamming algorithm based on DQN is proposed. The main research approach is outlined as follows:

(a) In the frequency domain, DRL is employed for intelligent frequency decision-making to select channels with the least interference. This approach can effectively avoid jamming, exhibits strong anti-jamming performance, and significantly improves communication quality. This strategy is not only applicable in static jamming environments but also remains effective under dynamically changing jamming conditions.
(b) In terms of transmission power, multiple transmission power levels are defined within the power domain. The configuration of these levels allows for flexible adjustment under varying environmental conditions. Higher power levels correspond to increased transmission power, which enhances anti-jamming capability at the cost of higher energy consumption. Therefore, it is crucial to rationally control the selection of transmission power while ensuring the accuracy and stability of data transmission. The objective is to meet communication quality requirements while minimizing energy consumption, thereby extending the UAV’s operational endurance and improving overall mission effectiveness.
(c) Regarding the transmission rate in the code domain, modulation and demodulation are jointly considered. Different modulation orders support different data transmission rates: higher modulation orders enable more data to be transmitted per unit of time, thereby increasing the data transmission rate. However, higher-order modulation schemes require better signal quality and are more susceptible to interference during transmission. Hence, maximizing the modulation order without compromising data correctness is key to improving UAV communication efficiency. This requires the UAV to intelligently select the most suitable modulation scheme based on the current communication environment and jamming conditions. In environments with high signal quality and low interference, higher-order modulation can be adopted to increase the data transmission rate; in environments with strong interference, the modulation order should be reduced to ensure reliable signal transmission. Through this adaptive strategy, both the data transmission rate and the stability and reliability of communication can be optimized across varying conditions, thereby enhancing the efficiency of UAV mission execution.
(d) Hierarchical reinforcement learning (HRL) is introduced, which decomposes complex decision-making problems into smaller, more manageable sub-tasks. Each sub-task is handled by an individual RL agent. This approach can accelerate the convergence of the learning process and enhance the anti-jamming capability of UAVs in complex environments.

#### 3.2.1. DQN-Based Anti-Jamming Decision-Making Algorithm

Under the RL framework, the MDP provides a mathematical model for decision-making by the agent. An MDP consists of five key elements: a state space (*S*), an action space (*A*), a state transition probability function (*P*), a discount factor (γ), and a reward function (*R*). These can be abstracted as the tuple (*S*, *A*, *P*, γ, *R*). In this paper, we model the scenario of point-to-point communication under unknown jamming as an MDP. In this model, the UAV acts as an agent that must make decisions in a dynamically changing jamming environment to ensure communication reliability and efficiency. The specific MDP model proposed in this paper is described as follows:

(a) State Space: The spectrum state is sensed using the *energy detection method* to evaluate the communication quality of each channel. In this process, the state of each channel is initialized to 0, indicating that all channels are initially assumed to be free of interference. If noise or jamming is detected in a channel, its state is marked as 1. Assuming the total number of channels is *c*, the size of the state space is $2^{c}$, reflecting all possible combinations of channel states.
(b) Action Space: The available actions include channel selection, transmission power, and modulation scheme. An action at step *k* is represented as $A_{k}=\left(C_{k},P_{k},M_{k}\right)$, where C=1,2,3,…,c denotes the set of available channels, P=1,2,…,p represents the power levels, M=1,2,…,m denotes the modulation levels, and *k* indicates the $k^{\mathrm{th}}$ group of transmission data. The size of the action space is defined as c×p×m.
(c) State Transition Probability: The state transition probability is denoted as P:S×A×S→[0,1], which defines the probability of transitioning to state $s_{k+1}$ given the current state ($s_{k}\in S$) and an action ($a_{k}\in A$). In this model, the state transition is assumed to be deterministic, meaning that for a given state and action, the next state is uniquely determined without randomness.
(d) Discount Factor: The discount factor satisfies 0<γ≤1 and determines the importance the agent assigns to future rewards. A value of γ closer to 1 indicates higher importance placed on long-term rewards, while a smaller γ implies a greater emphasis on short-term rewards.
(e) Reward Function: At time step *t*, the BER is calculated after data transmission. If the BER is less than $10^{-5}$, the data transmission is considered successful, and the reward value is given by Equation (3):
  (3) R=1+α·power+β·modulation.
  In Equation (3), power denotes the transmission power level; modulation denotes the modulation order level; and α and β represent the weight coefficients for the transmission power level and modulation order level, respectively, with α>β. The purpose of this design is to prioritize guaranteeing the data transmission rate by first adjusting the transmission power, then the modulation order. If the BER exceeds $10^{-5}$, the data transmission is considered erroneous, and the reward value is given by Equation (4):
  (4) R=0

#### 3.2.2. HRL-Based Anti-Jamming Decision-Making

Since this study is based on the frequency domain, power domain, and code domain, although it can reduce energy consumption and improve transmission rate, the large action space leads to a slow convergence speed of the algorithm. To enhance the convergence speed of the algorithm and improve anti-jamming capability, this study proposes an anti-jamming model based on a HRL framework, providing effective jamming management strategies for communication in UAV swarm systems. HRL decomposes complex decision-making processes into multiple sub-tasks [17], each controlled by a sub-policy, thereby breaking down a large action space into several smaller and more manageable sub-spaces.

**Figure 4:**
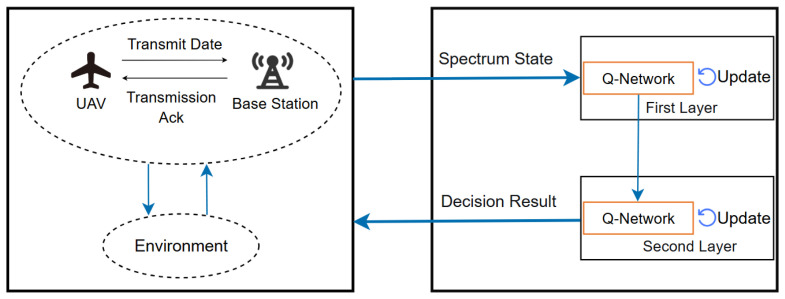
Process framework of the anti-jamming method based on HRL.

The model decomposes the RL process into two main layers: the first layer is the frequency domain layer, and the second layer is the power and code domain layer. Channels serve as the fundamental medium of communication. Prioritizing the selection of low-interference channels reduces the complexity of subsequent power and modulation adjustments. If power were adjusted first, excessive channel interference could lead to power wastage. Therefore, the hierarchical sequence aligns with the decision-making logic of addressing fundamentals before optimizations. This enables detailed optimization of communication strategies, ensuring that UAVs transmit data in the best possible communication environment while conserving energy and increasing transmission rates. The procedural framework of the HRL-based anti-jamming method is shown in Figure 4.

In the design of this model, the DQN process is divided into two layers. The first layer focuses on channel selection, with the key task of identifying and selecting an optimal communication path, i.e., the channel with the least noise and interference. Upon proceeding to the second layer, the channel selection decision made in the frequency-domain layer serves as critical state input, providing the necessary contextual information for the agent to adjust transmission power and select appropriate modulation schemes accordingly. Such a hierarchical design ensures that the overall communication strategy is developed under the optimal or most suitable environmental conditions, significantly enhancing communication efficiency and quality.

Through this approach, the decision-making process for communication strategies is ensured to be hierarchical and logical. It first establishes the fundamental conditions in the frequency-domain layer, then further refines and optimizes these in the power- and code-domain layers. This not only enhances communication reliability but also improves energy efficiency, providing an effective anti-jamming communication solution for UAV swarm systems in complex interference environments.

The MDP model for the first layer of the HRL framework proposed in this paper is specified as follows:

**(a)**  **State Space:** The current state represents the spectrum status, sensed via the energy detection method to determine channel conditions. The state of all channels is initialized to 0, where a value of 1 indicates that the current channel is affected by noise or interference and 0 is the value otherwise. Given that there are *c* channels in total, the size of the state space is $2^{c}$.
**(b)**  **Action Space:** The action involves selecting an available channel. Let C=1,2,3,…,c denote the set of available channels, where *c* is the total number of selectable channels. The size of the action space is defined as *c*.
**(c)**  **State Transition Probability:** The state transition probability is denoted as P:S×A×S→[0,1].
**(d)**  **Discount Factor:** The discount factor satisfies 0<γ≤1.
**(e)**  **Reward Function:** The reward function for the first layer is given by Equation (5):
  (5) $$R=\left\{\begin{array}{cc} 1, & \mathrm{if}\;\mathrm{BER}<10^{-5}\;(\mathrm{transmission}\;\mathrm{successful})\;;\\ 0, & \mathrm{otherwise}\;(\mathrm{transmission}\;\mathrm{failed})\;. \end{array}\right.$$
  In Equation (5), at time step *t*, the BER is calculated after data transmission. If the BER is less than $10^{-5}$, the data transmission is considered correct and the reward value is 1; otherwise, the reward value is 0.

The MDP model for the second layer of the HRL framework proposed in this paper is specified as follows:

**(a)**  **State Space:** The state is defined as the communication channel selected by the first layer. All channels are initialized to 0, and the selected communication channel is set to 1. Given that there are *c* channels in total, the size of the state space is $2^{c}$.
**(b)**  **Action Space:** The action involves selecting a transmission power and modulation scheme. Let P=1,2,…,p denote the power levels, where *p* represents the maximum power level, and let M=1,2,…,m denote the modulation levels, where *m* represents the maximum modulation level. The size of the action space is defined as p×m.
**(c)**  **State Transition Probability:** The state transition probability is denoted as P:S×A×S→[0,1].
**(d)**  **Discount Factor:** The discount factor satisfies 0<γ≤1.
**(e)**  **Reward Function:** The reward function for the second layer is given by Equation (6):
  (6) $$R=\left\{\begin{array}{cc} \alpha \times \mathrm{power}+\beta \times \mathrm{modulation}, & \mathrm{if}\;\mathrm{BER}<10^{-5}\;(\mathrm{transmission}\;\mathrm{successful})\;;\\ 0, & \mathrm{otherwise}\;(\mathrm{transmission}\;\mathrm{failed})\;. \end{array}\right.$$
  In Equation (6), power denotes the transmission power level; modulation denotes the modulation order level; and α and β represent the weight coefficients for the transmission power level and modulation order level, respectively, with α>β. The purpose of this design is to prioritize guaranteeing the data transmission rate by first adjusting the transmission power and, then the modulation order. If the BER is less than $10^{-5}$, the transmission is considered successful and the reward value is α×power+β×modulation; otherwise, the reward value is 0.

#### 3.2.3. Algorithm Flow

The intelligent anti-jamming decision-making process based on multi-domain collaborative DQN proceeds as follows. Initially, the evaluation network and target network are established, and the experience replay buffer is initialized, along with the parameters. At the beginning of each time slot, the UAV performs spectrum sensing and determines the optimal channel, transmission power, and modulation scheme based on the sensing results. Communication is then carried out through the communication system, and a reward value is returned according to the transmission outcome. This process repeats until the maximum number of iterations is reached.

**Figure 5:**
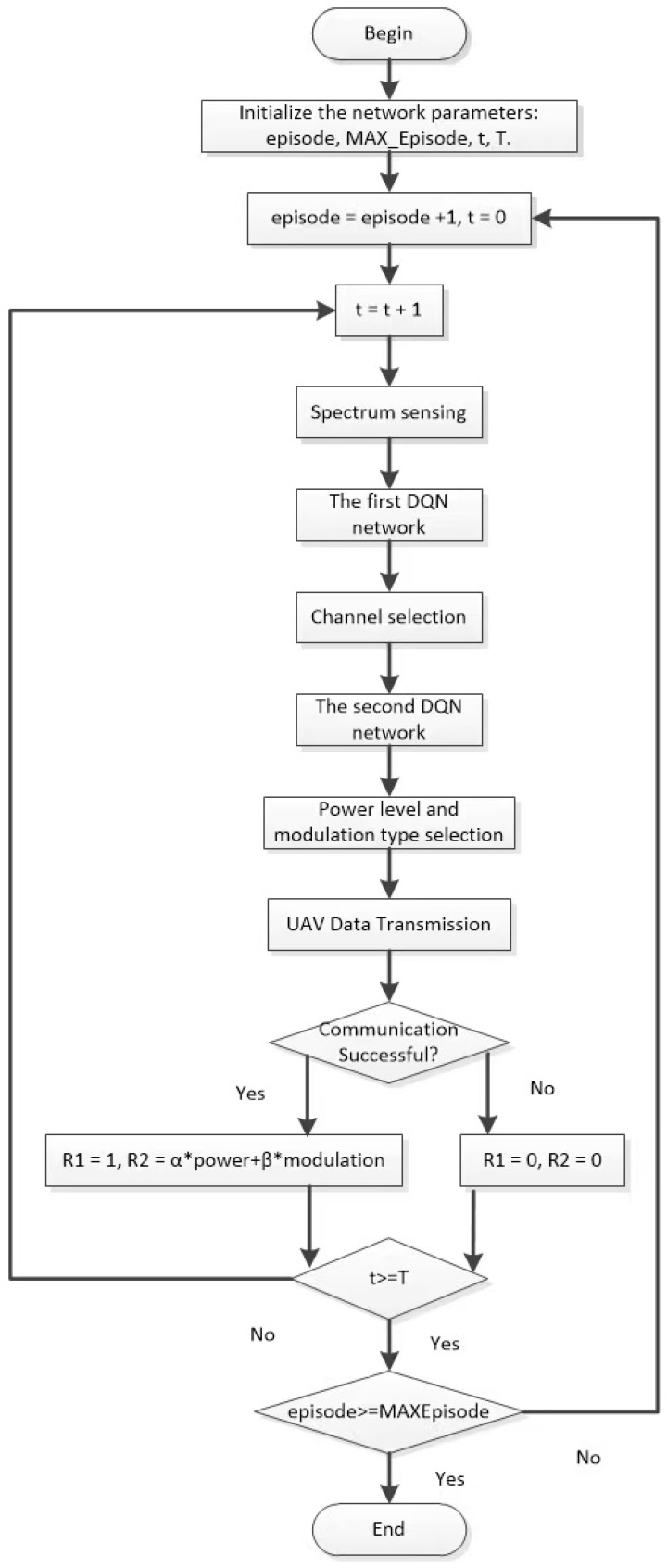
Framework of the anti-jamming method based on HRL.

The process of the intelligent anti-jamming decision-making method based on HRL proceeds as follows. In this process, a hierarchical DQN is constructed to optimize the UAV’s communication strategy. The first-level DQN is responsible for selecting the optimal transmission channel, while the second-level DQN determines the transmission power and modulation–demodulation scheme based on the selected channel. This is followed by data transmission through the communication system. Depending on the success or failure of the communication, the reward values of both DQN levels are updated accordingly to reflect the learning outcomes. This process continues through multiple interactions with the communication environment until the predefined maximum number of updates is reached. Through this approach, the UAV can adapt to dynamically changing communication environments, effectively counter various interferences, and ensure efficient and reliable communication. The overall procedure of the hierarchical reinforcement learning algorithm is shown in Figure 5.

## 4. Results

### 4.1. Experimental Settings

The simulation environment employs the PyTorch 1.12.0 deep learning framework and the MATLAB R2021a simulation platform.

The model parameter settings are shown in Table 2. The simulation considers the following four jamming patterns:

1. Single-tone jamming: Channel 1 is continuously jammed.
2. Multi-tone jamming: Channels 1, 3, 5, 7, and 9 are continuously jammed.
3. Tracking jamming: If the channel selected in the current time slot is the same as that in the previous time slot, it is guaranteed to be jammed. If the selected channel is one of the two channels adjacent to the previously selected channels, it has a 0.5 probability of being jammed.
4. Sweep jamming: Starting from channel 1, the jamming channel switches sequentially each time slot, creating periodic interference.

The communication system employs an OFDM system with simulation parameters based on the IEEE 802.11a WLAN PHY layer standard. Specific parameters are listed in Table 3, while the transmission rates corresponding to modulation schemes are provided in Table 4. The jamming and communication environments are generated by a MATLAB-based simulation platform. The performance is evaluated based on four primary metrics: the convergence of reward values is used to assess model convergence, while the communication success rate, average power level, and average transmission rate are employed to evaluate the model’s anti-jamming performance. The study presented in this paper considers a dynamic environment where no specific altitude is predetermined; however, the scenario assumes favorable channel conditions typically encountered at higher altitudes. The communication model involves a single pair of UAVs, as illustrated by the two-node model in Figure 1.

### 4.2. Analysis of Experimental Results

To validate the effectiveness of the algorithm proposed in this paper, simulations were conducted on the algorithm’s convergence, transmission success rate, average data rate, and average power level. This study selects five methods for comparison with the proposed KG-DQN: the multi-domain joint cognitive anti-jamming intelligent decision-making algorithm based on deep double QL network (TGDJ-AJ) proposed in [18] and the novel anti-jamming algorithm based on PER-DQN (JAJA-PDQN) proposed in [19], along with the commonly used QL and random selection methods, to verify the performance of the proposed algorithm.

#### 4.2.1. Convergence

The convergence of the algorithm is reflected by the reward values. The reward values under four types of jamming are shown below, with the reward value of KG-DQN taken as the average of the two-layer rewards.

**Figure 6:**
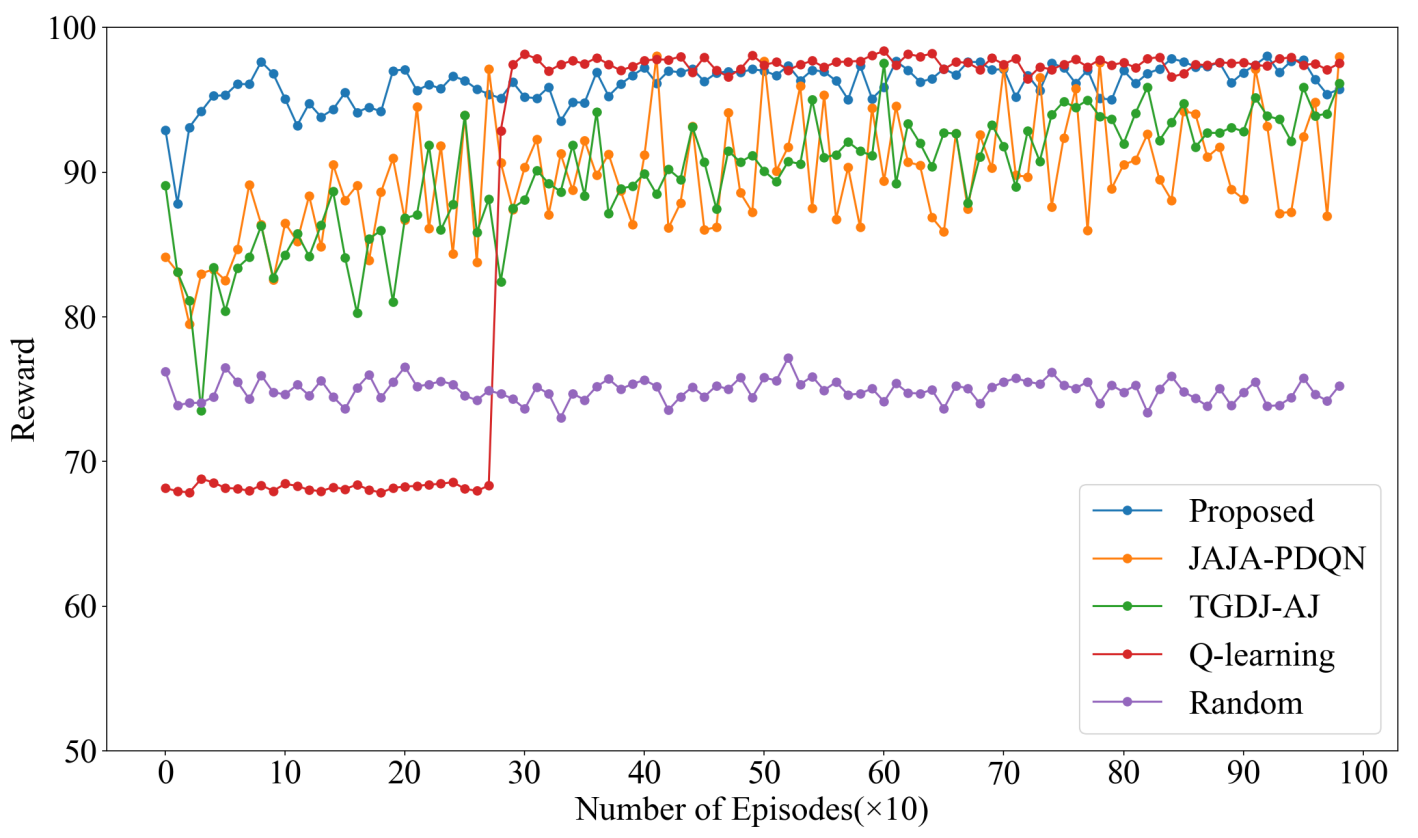
Reward values under single-tone jamming.

The reward values under single-tone jamming are shown in Figure 6. The results indicate that KG-DQN converges within 10 episodes, whereas JAJA-PDQN and TGDJ-AJ both converge around 200 episodes. Due to the lack of deep learning-based feature extraction and generalization capabilities, the traditional QL method requires approximately 300 episodes to reach the same convergence level. The reward value under the random method remains around 75. Compared to KG-DQN, the post-convergence reward values of JAJA-PDQN and TGDJ-AJ are significantly lower, and the reward value of JAJA-PDQN exhibits noticeably larger fluctuations after convergence.

**Figure 7:**
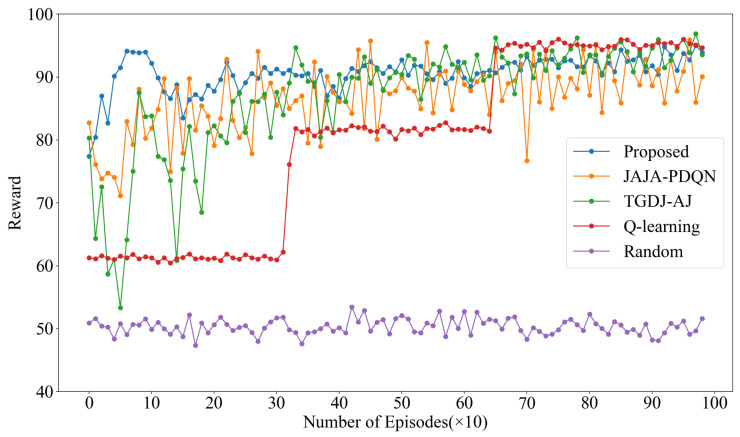
Reward values under multi-tone jamming.

The reward values under multi-tone jamming are shown in Figure 7. KG-DQN converges within 100 episodes, while JAJA-PDQN and TGDJ-AJ converge around 200 episodes. In contrast, QL requires approximately 650 episodes to reach convergence, and the reward value under the random method remains around 50. The results demonstrate that KG-DQN exhibits favorable convergence performance under multi-tone jamming. Although QL achieves relatively high reward values after convergence—attributed to its direct value lookup from the Q-table, which provides precise action-value estimates if the table is well-converged—its primary drawback is a significantly slower convergence speed.

**Figure 8:**
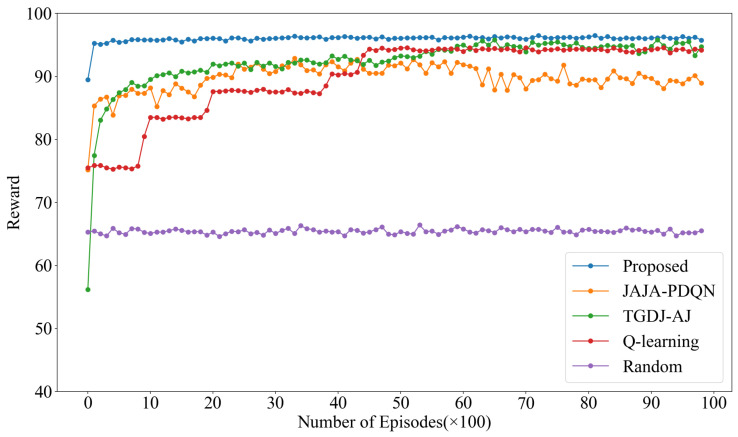
Reward values under tracking jamming.

The reward values under tracking jamming are shown in Figure 8. KG-DQN achieves convergence within 10 episodes, while JAJA-PDQN and TGDJ-AJ converge in approximately 100 episodes. In comparison, QL requires about 500 episodes to reach convergence, and the reward value under the random method fluctuates around 65. The reward value of KG-DQN is significantly higher than that of the other algorithms, indicating that KG-DQN demonstrates superior performance in relatively complex jamming environments.

**Figure 9:**
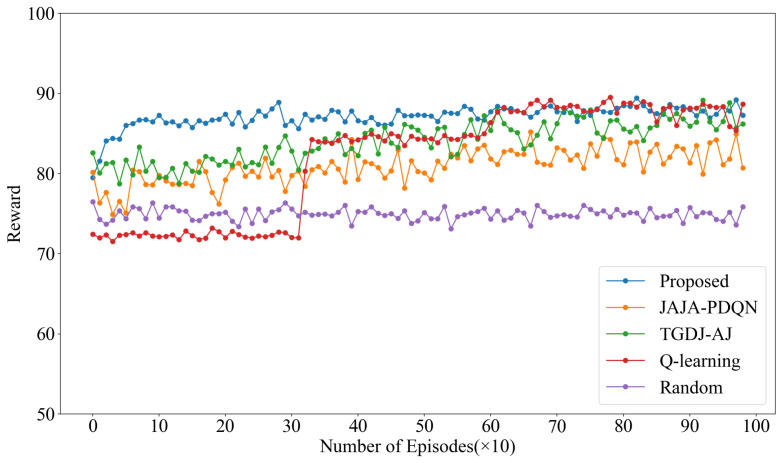
Reward values under sweep jamming.

The reward values under sweep jamming are shown in Figure 9. KG-DQN achieves convergence within 100 episodes, while JAJA-PDQN and TGDJ-AJ converge in approximately 300 episodes. In contrast, QL requires about 600 episodes to reach convergence, and the reward value under the random method fluctuates around 75.

The hierarchical DQN architecture decouples the multi-dimensional parameter optimization problem into a two-level optimization of two-dimensional parameters, thereby enhancing the convergence speed.

Compared to the other three jamming types, the post-convergence reward values under sweep jamming are lower. This is due to the wide frequency coverage of sweep jamming, which makes it difficult for the UAV to identify patterns and select channels with less interference for communication, consequently increasing the probability of communication interruptions and data transmission failures.

#### 4.2.2. Communication Success Rate

**Figure 10:**
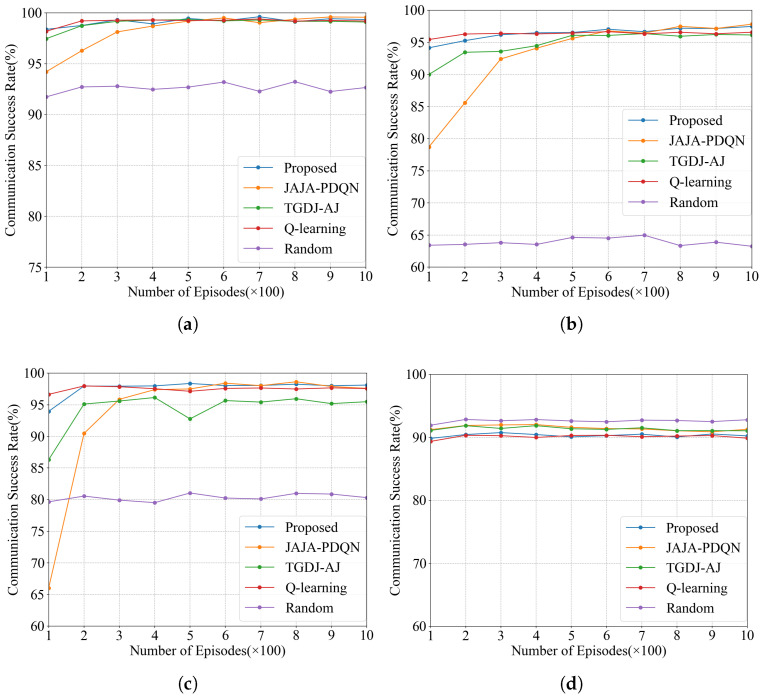
Communication success rate under four types of jamming: (**a**) communication success rate under single-tone jamming; (**b**) communication success rate under multi-tone jamming; (**c**) communication success rate under tracking jamming; (**d**) communication success rate under sweep jamming.

The communication success rates under the four types of jamming are shown in Figure 10. The convergence speed of the KG-DQN algorithm in terms of communication success rate is significantly faster than that of the JAJA-PDQN and TGDJ-AJ algorithms.

The following analyzes the experimental results over 1000 episodes. Under single-tone jamming, the KG-DQN algorithm improves the communication success rate by 0.7% over the JAJA-PDQN algorithm. Under multi-tone jamming, the KG-DQN algorithm improves the communication success rate by 3.4% over the JAJA-PDQN algorithm. Under tracking jamming, the KG-DQN algorithm improves the communication success rate by 4.2% over the JAJA-PDQN algorithm. Under sweep jamming, the KG-DQN algorithm attains a slightly lower communication success rate than the random method, which is attributed to the random method’s greater effectiveness in avoiding this type of wide-area periodic interference.The effectiveness of the proposed method in improving the communication success rate has been validated.

#### 4.2.3. Average Power Level

**Figure 11:**
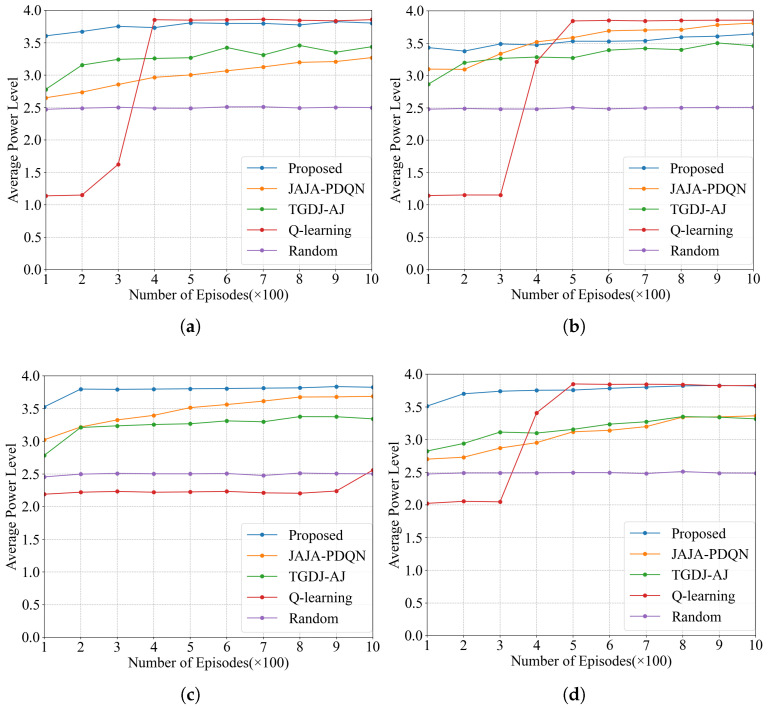
Average power level under four types of jamming: (**a**) average power level under single-tone jamming; (**b**) average power level under multi-tone jamming; (**c**) average power level under tracking jamming; (**d**) average power level under sweep jamming.

The average power levels under the four types of jamming are shown in Figure 11. After convergence, the power level of KG-DQN under all four jamming types is significantly higher than that of JAJA-PDQN, TGDJ-AJ, and the random method. KG-DQN demonstrates strong performance under the relatively complex tracking jamming scenario, and its average power level also converges the fastest. Although under multi-tone jamming, the final average power level of KG-DQN is slightly lower than that of QL, KG-DQN converges much faster. Furthermore, the average power level of the QL method under tracking jamming is even lower than that of the random method. Therefore, overall, the KG-DQN method exhibits superior performance.

The average power levels of different anti-jamming methods over 1000 episodes are shown in Table 5. Under all four jamming types, the average power level of KG-DQN is significantly higher than that of other methods, remaining above 3.5, while the highest average power level among the remaining methods is only 3.3.

#### 4.2.4. Average Transmission Rate

**Figure 12:**
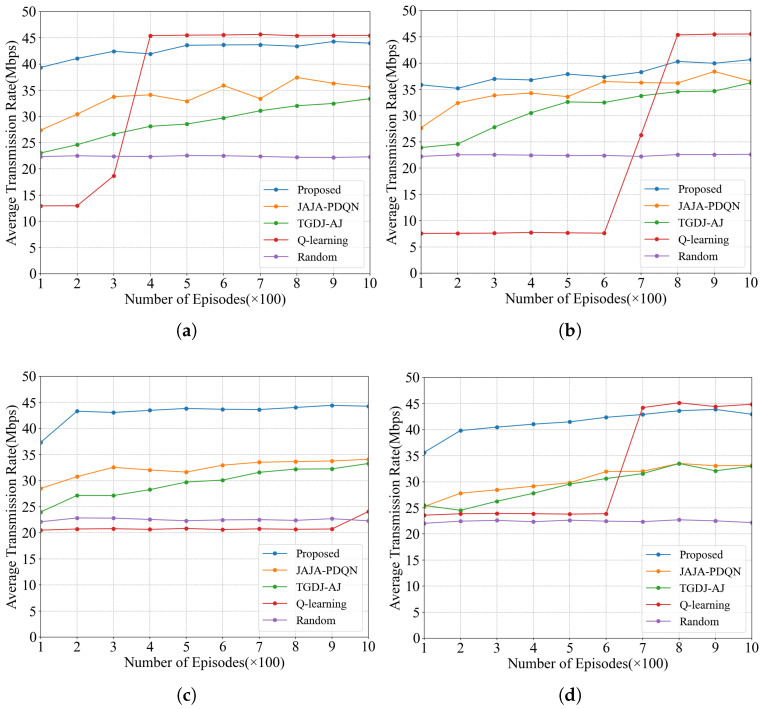
Average transmission rate under four types of jamming: (**a**) average transmission rate under single-tone jamming; (**b**) average transmission rate under multi-tone jamming; (**c**) average transmission rate under tracking jamming; (**d**) average transmission rate under sweep jamming.

The average transmission rates under the four types of jamming are shown in Figure 12. Similarly, the average transmission rates of JAJA-PDQN and TGDJ-AJ remain relatively low across all four jamming scenarios. KG-DQN demonstrates excellent adaptability when handling complex tracking jamming scenarios while also leading other methods in convergence speed. The average transmission rates of different anti-jamming methods over 1000 episodes are shown in Table 6; KG-DQN achieves a significantly faster average transmission rate compared to other methods.

The following analyzes the experimental results over 1000 episodes. Under single-tone jamming, the KG-DQN algorithm improves the average transmission rate by 47.6% over the TGDJ-AJ algorithm. Under multi-tone jamming, the KG-DQN algorithm improves the average transmission rate by 81.8% over the QL algorithm. Under tracking jamming, the KG-DQN algorithm improves the average transmission rate by 104.8% over the QL algorithm. Under sweep jamming, the KG-DQN algorithm improves the average transmission rate by 40.7% over the TGDJ-AJ algorithm. The effectiveness of the proposed method in improving the transmission rate has been validated.

Simulation results validate the superiority of the two-stage intelligent anti-jamming decision-making method based on KG and HRL proposed in this paper. Compared to existing methods such as JAJA-PDQN, TGDJ-AJ, QL, and the random method, our approach demonstrates significant advantages across multiple key performance metrics, including convergence speed, communication success rate, average power level, and average transmission rate.

First, regarding convergence performance, the proposed method achieves much faster convergence under four typical jamming scenarios—single-tone, multi-tone, tracking, and sweep jamming—compared to the baseline algorithms. This can be primarily attributed to two core design elements: Firstly, the knowledge graph repository provides rapid reasoning and initial decision-making capabilities, effectively avoiding blind exploration by the RL agent during the initial phase. Secondly, the HRL architecture decomposes the high-dimensional action space (encompassing channel, power, and modulation) into two lower-dimensional sequential decision-making processes (channel selection and power and modulation selection). This significantly reduces the exploration complexity of the algorithm, thereby accelerating convergence.

Second, concerning anti-jamming effectiveness and resource utilization, all intelligent methods achieve high communication success rates after convergence under relatively simple single-tone and multi-tone jamming. However, under the more complex tracking and sweep-jamming scenarios, our method maintains superior performance in communication success rate, average power level, and transmission rate, leveraging its hierarchical decision-making structure and dynamic interactive learning capability. This indicates that the method not only effectively avoids jamming but also intelligently coordinates multi-domain resources: based on channel selection for jamming avoidance, it tends to select higher transmission power and higher-order modulation schemes. Consequently, it enhances data transmission efficiency and the system’s jamming resilience while ensuring communication reliability. Particularly under sweep jamming, a type of full-band periodic interference where the performance of all methods degrades, our method still maintains better performance than most comparative algorithms through adaptive adjustment of power and modulation, demonstrating the robustness and adaptability of its strategy.

Finally, this study has several limitations that warrant further investigation. While the model has been effectively validated in a single-UAV scenario, extending the framework to multi-UAV collaborative anti-jamming communication scenarios and addressing the associated non-stationarity issues represent an important direction for future research. Furthermore, although the proposed method achieves anti-jamming success rates of over 90% against common types of jamming, its performance in mitigating sweep jamming remains suboptimal, with lower communication success rates compared to other jamming types. Therefore, exploring and developing more effective strategies to enhance communication reliability under sweep jamming—such as predicting its frequency-hopping patterns for proactive avoidance—constitutes another critical area for future improvement.

## 5. Conclusions

To address the challenge of rapidly identifying jamming signals and selecting optimal anti-jamming strategies in complex electromagnetic environments, this paper proposes a novel two-stage intelligent anti-jamming decision-making framework integrating a KG and a DQN. Theoretical analysis and simulation results demonstrate the following key achievements:

An anti-jamming KG repository was constructed by manually extracting domain knowledge, encompassing entities such as jamming patterns, anti-jamming strategies, time-frequency features, and decision parameters. Utilizing RDF triples for knowledge representation and storing the graph in a Neo4j database enabled efficient knowledge storage and reasoning. This provides the system with a structured knowledge base capable of fast response.

A dynamic optimization method based on HRL was proposed to tackle the convergence issue. By decomposing the complex multi-dimensional joint decision-making problem into a two-layer hierarchy—“channel selection” followed by “power and modulation selection”—the action space was significantly reduced, thereby lowering the algorithm’s complexity. Simulation results confirm that this hierarchical architecture greatly accelerates convergence. It allows the model to learn effective policies across various jamming scenarios, ensuring high communication success rates while enabling efficient utilization of resources like transmission power and data rates.

In summary, the proposed KG-DQN intelligent anti-jamming framework combines both real-time responsiveness and adaptability, offering an effective solution for achieving reliable communication in complex and dynamic electromagnetic environments.

Although the proposed model demonstrates superior anti-jamming performance and convergence characteristics, it does not consider decision consistency in multi-node networking scenarios, particularly the consistency of frequency usage decisions. In future work, we will employ federated learning to conduct distributed training of multi-node decision models, thereby ensuring both consistency in frequency usage decisions and data security.

## Figures and Tables

**Table 1:** Complete abbreviation mapping for knowledge graph entities.

Abbreviation	Full Term
**Jamming Patterns**	
STJ	Single-Tone Jamming
BBJ	Broadband Barrage Jamming
PBJ	Partial Band Jamming
MTJ	Multi-tone (Comb) Jamming
TRJ	Tracking Jamming
SWJ	Sweep Jamming
PPJ	Periodic Pulse Jamming
HSCJ	High-Speed Collision Jamming
MAJ	Modulation Aimed Jamming
CAJ	Code Aimed Jamming
CPKJ	CSMA Protocol Keyframe Jamming
**Anti-Jamming Strategies**	
DFA	Dynamic Frequency Adaptation
DPA	Dynamic Power Adaptation
DMCPA	Dynamic Modulation Coding Parameter Adaptation
IHR	Increase Hopping Rate
DHFSA	Dynamic Hopping Frequency Set Adaptation
DDTA	Dynamic Dwell-Time Adaptation
DPPA	Dynamic Protocol Parameter Adaptation
**Jamming Features**	
EFFG	Existence of Full-time Frequency Gaps
EIFG	Existence of Instantaneous Frequency Gaps
EPM	Existence of Power Margin
ETG	Existence of Time Gaps
**Decision Parameters**	
CF	Communication Frequency
CB	Communication Band
MCC	Modulation Coding Combination
DT	Dwell Time
TP	Transmit Power
KFD	Key Frame Delay
SS	Subcarrier Set
**Jamming Pattern Attributes**	
CFreq	Center Frequency
JP	Jamming Power
JSF	Jamming Start Frequency
JF	Jamming Frequency
TB	Total Bandwidth
IB	Instantaneous Bandwidth
JPer	Jamming Period
JFS	Jamming Frequency Set
IMAJ	Is Modulation Aimed Jamming
ICAJ	Is Code Aimed Jamming
ICPKJ	Is CSMA Protocol Keyframe Jamming
TPow	Total Power
EF	End Frequency
SPP	Single Point Power
IP	Instantaneous Power
TRate	Tracking Rate
DC	Duty Cycle

**Table 2:** Parameter settings for the single-UAV anti-jamming model.

Parameter	Assigned Value
Epsilon (greedy rate)	0.9
Lambda (reward decay rate)	0.9
Alpha (learning rate)	0.01
Memory capacity	10,000
Slots per episode	100
c (channel count)	10
p (power level)	4
m (modulation level)	4
α (weight)	0.15
β (weight)	0.1

**Table 3:** OFDM simulation parameter settings.

Parameter	Assigned Value
Modulation Type	BPSK, QPSK, 16QAM, 64QAM
Nimber of Subcarriers	52
Number of Pilots	4
OFDM Symbol Length (μs)	4
Guard Interval Length (ns)	800
Subcarrier Spacing (kHz)	312.5
Signal Bandwidth (MHz)	16.66
Channel Spacing (MHz)	20

**Table 4:** Data transfer rates corresponding to modulation methods.

Modulation Method	Data Transfer Rate (Mbps)
BPSK	6
QPSK	12
16QAM	24
64QAM	48

**Table 5:** Average power levels of different anti-jamming methods over 1000 episodes.

Anti-Jamming Method	Single-Tone Jamming	Multi-Tone Jamming	Tracking Jamming	Sweep Jamming
Proposed	3.8	3.5	3.8	3.7
JAJA-PDQN	3.4	3.4	3.5	3.4
TGDJ-AJ	3.2	3.3	3.2	3.2
Q-learning	3.1	3.0	2.3	3.3
Random	2.5	2.5	2.5	2.5

**Table 6:** Average transmission rates of different anti-jamming methods over 1000 episodes.

Anti-Jamming Method	Single-Tone Jamming	Multi-Tone Jamming	Tracking Jamming	Sweep Jamming
Proposed	42.79	37.99	43.16	41.46
JAJA-PDQN	33.77	34.63	32.39	30.46
TGDJ-AJ	28.99	31.17	29.61	29.46
Q-learning	36.35	20.90	21.07	32.21
Random	22.40	22.49	22.53	22.45

## Data Availability

The data supporting the findings of this study are not publicly available due to confidentiality restrictions. Requests for access to the data may be directed to the corresponding author, but availability cannot be guaranteed.

## Abbreviations

The following abbreviations are used in this manuscript:

QL	Q-Learning
MDP	Markov Decision Process
RL	Reinforcement Learning
DRL	Deep Reinforcement Learning
KG	Knowledge Graph
HRL	Hierarchical Reinforcement Learning
DQN	Deep Q-network
UAV	Unmanned Aerial Vehicle
BER	Bit Error Rate
KG-DQN	Knowledge Graph-Deep Q-Network
RDF	Resource Description Framework
JSR	Jamming-to-Signal Ratio

## References

[1] Zhou Q., Niu Y., Xiang P., Li Y. (2023). Intra-domain knowledge reuse assisted reinforcement learning for fast anti-jamming communication. IEEE Trans. Inf. Forensics Secur..

[2] Zhang X., Wang H., Ruan L., Xu Y., Feng Z. Joint channel, power and bandwidth optimization for anti-jamming communications: A multi-agent Q-learning approach. Proceedings of the 2021 13th International Conference on Wireless Communications and Signal Processing (WCSP).

[3] Niu Y., Feng X., Kou S., Xiang P. (2022). A novel anti-jamming decision-making algorithm based on knowledge graph technology. Appl. Sci..

[4] Zhou Q., Li Y., Niu Y. (2021). Intelligent anti-jamming communication for wireless sensor networks: A multi-agent reinforcement learning approach. IEEE Open J. Commun. Soc..

[5] Chen C., Song M., Xin C., Backens J. (2013). A game-theoretical anti-jamming scheme for cognitive radio networks. IEEE Netw..

[6] Feng X., Niu Y., Liu Q., Zhou Q. (2024). Decision making for communication anti-jamming tasks with knowledge-graph-based Q-learning. Electronics.

[7] Zhang X. (2019). Research on Anti-Jamming Methods for Unmanned Aerial Vehicle Networks. Ph.D. Thesis.

[8] Jia L., Xu Y., Sun Y., Feng S., Yu L., Anpalagan A. (2018). A multi-domain anti-jamming defense scheme in heterogeneous wireless networks. IEEE Access.

[9] Xu Y., Ren G., Chen J., Luo Y., Jia L., Liu X. (2018). A one-leader multi-follower Bayesian-Stackelberg game for anti-jamming transmission in UAV communication networks. IEEE Access.

[10] Yang D., Zhang J., Fang X., Richa A., Xue G. Optimal transmission power control in the presence of a smart jammer. Proceedings of the 2012 IEEE Global Communications Conference (GLOBECOM).

[11] Yang D., Zhang J., Fang X., Richa A., Xue G. (2013). Coping with a smart jammer in wireless networks: A Stackelberg game approach. IEEE Trans. Wireless Commun..

[12] Li M., Ren Q., Wu J. (2021). Exploring UAV’s multi-domain joint anti-jamming intelligent decision algorithm. J. Northwestern Polytech. Univ..

[13] Liu C., Liu M., Ding Y. (2020). Cognitive anti-jamming algorithm for UAV cluster based on multiple domain combination. Comput. Eng..

[14] Zhang X., Wang H., Ruan L., Xu Y., Feng Z. (2022). Joint channel and power optimisation for multi-user anti-jamming communications: A dual mode Q-learning approach. IET Commun..

[15] Yin Z., Lin Y., Zhang Y., Qian Y., Shu F., Li J. (2022). Collaborative multiagent reinforcement learning aided resource allocation for UAV anti-jamming communication. IEEE Internet Things J..

[16] Adamy D. (2015). EW 104: Electronic Warfare Against a New Generation of Threats.

[17] Pateria S., Subagdja B., Tan A.H., Quek C. (2021). Hierarchical reinforcement learning: A comprehensive survey. ACM Comput. Surv..

[18] Zhang H.T., Zhang R., Liu M.T., Ding Y.M. (2022). Deep reinforcement learning-based anti-jamming algorithm for UAV communication. J. Ordnance Equip. Eng..

[19] Wan B., Niu Y., Chen C., Zhou Z., Xiang P. (2023). A novel algorithm of joint frequency-power domain anti-jamming based on PER-DQN. Neural Comput. Appl..

